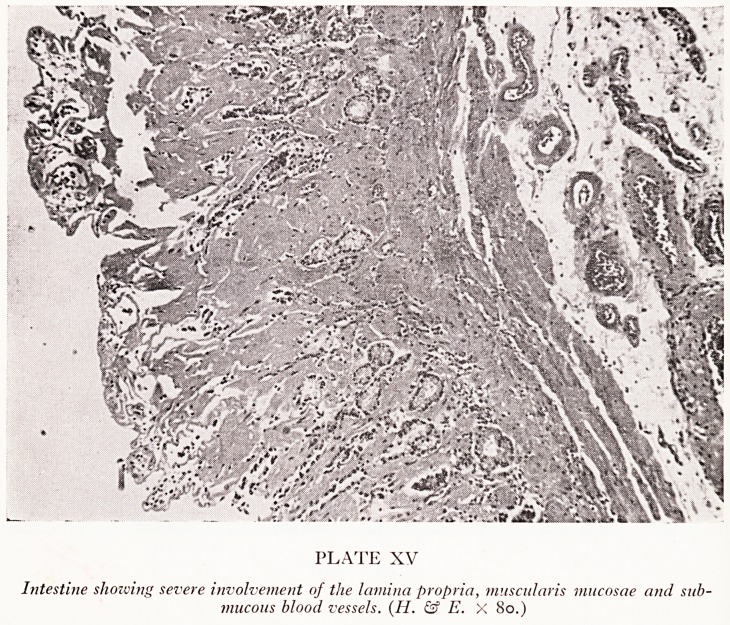# Primary Systemic Amyloidosis

**Published:** 1964-07

**Authors:** 


### PRIMARY SYSTEMIC AMYLOIDOSIS

*Clinico-Pathological Conference held on 25th February* 1964

(*P.M. No.* 8133).

*Dr. Harrold:* This man was 68 when he died. He was a gentlemen's outfitter. Before this illness he had had two hernia repairs, and during the 1914-1918 war he had had a few injuries to the arms and legs. He had also had a brief episode of jaundice.

His final illness began in the summer of 1962. He died in the summer of 1963. In 1962 he noticed the onset of progressive mild exertional dyspnoea. In November 1962 he went to see his doctor because he had noticed a blown-up feeling in his abdomen, which was mildly painful. His doctor referred him to Mr. Paul, who did not find any physical abnormality and arranged for him to have a barium enema because he gave a history of slight irregularity of bowel habit. The enema was normal. He was then given a barium meal because of the pain in his abdomen, and this again was normal. However, the man looked ill and Mr. Paul suggested to the general practitioner that a physician's opinion should be sought.

When Dr. Cates saw this man a month later he was jaundiced and had an epigastric mass. His clinical state suggested that there might be abdominal malignancy. He was admitted to the B.R.I. for laparotomy. When he came in the houseman taking his history discovered that he had lost 2-3 stones in weight during the last few months, and that on one or two days before admission he had been vomiting. For some months he had been anorexic. He looked pale and ill and had a raised jugular venous pressure, which had not been noticed before. He had basal râles and an even larger mass in the abdomen. It was described as liver and extended to 5 fingers breadth below the costal margin. Nevertheless there was still an epigastric mass which seemed distinct from the liver.

At laparotomy, one week after admission, he was seen to have a large, congested, irregularly nodular liver, the nodularity involving both lobes. There was no evidence of neoplasm in the stomach, pancreas, duodenum, large or small bowel, kidneys or bladder. A liver biopsy showed amyloidosis.

Two weeks after laparotomy his abdominal wound burst and had to be resutured, after which the wound healed very well. He then made a reasonably good recovery. Some weeks later, on 6th March, he suddenly felt short of breath after taking a bath. On examination he was found to have an irregular pulse which was about 90/min, his blood pressure had fallen to 95/60. An ECG showed atrial tachycardia with atrio-ventricular block, so that the atrial rate was about 160/min and the ventricular rate was irregular and about 90/min. After this he developed increasing oedema, ascites and breathlessness. His jaundice persisted and he was transferred to the medical ward.

When I first saw him he was very ill, very depressed and unhappy and I thought he had obvious insight into the hopeless prognosis of his condition. He was jaundiced, had marked engorgement of the neck veins, he had gross pitting oedema involving the legs and body up to the level of the lower chest. He had a weak pulse which was irregular, his heart was enlarged, the apex beat was felt in the anterior axillary line, the lung bases were dull to percussion and the breath sounds were reduced. He had gross abdominal swelling and the physical signs of ascites. There was a fluid thrill. Because of the ascites the liver was not easily palpated but it could be felt by deep and rather painful pressure on the abdomen. We gave him digoxin and a diuretic, chlorthiazide, and nothing happened except that his ventricular rate was a little better controlled than it had been. So we added two other drugs, aldactone, which is an aldosterone antagonist and acts as a diuretic agent, and triamterine, which is another diuretic agent which behaves in the same way as aldosterone antagonists but does not depend on the presence of aldosterone for its action. With these three drugs his oedema went. We were very pleased with this but nevertheless the man remained ill.

Knowing that he had amyloid disease we wondered how many organs might be involved. We had good evidence that his heart was involved; he had cardiomegaly and heart failure. We had no evidence that there was another cause for this heart failure so we thought we had good evidence of amyloid disease of the heart. We looked at his tongue, because this is often big in people with primary amyloidosis, and his tongue was normal. We had histological evidence that his liver was involved. The functional evidence of liver involvement was jaundice, the high alkaline phosphatase, 45 at its highest, bilirubin 4.85 mgm per cent, with the conjugated bilirubin a little more than the unconjugated, but both were increased. His serum proteins were not grossly abnormal although the serum albumen was low, its lowest figure being 2.5 gm per cent. The alpha-2 globulin was raised a little above the highest level of normal to 0·85 g per cent, the upper limit of normal being 0·7 g per cent. So we had good evidence that the liver was involved.

We wondered whether he had involvement of the kidneys. His urinary protein was measured by Esbach's method. This was based on the total 24-hour urinary output and occasionally we had figures of $\frac{1}{4}$ g, but I think this is an unreliable estimate of urinary protein output as we found none on boiling, and there was really no significant proteinuria. His blood urea varied between 19 and 40 mgm per cent. His centrifuged urinary deposit was normal. So we had no evidence that there was renal disease.

His spleen was not palpable. He was mildly anaemic; at its lowest his Hb was 76 per cent. There were mild toxic changes in the polymorphonuclear white cells. He could have had involvement of spleen or bone marrow or both. Whether or not his adrenals were involved is also a difficult question to answer. He had noticed that the moles on his skin had become a little more pigmented in recent months. He was hypotensive but we had another adequate explanation for this, in that he had severe myocardial disease. He had no oral pigmentation although he was of course jaundiced. He did not have the pigmentation of Addison's disease. We did not estimate his urinary steroid output, so we had no absolute estimate of suprarenal function. We wondered whether his peripheral nerves were involved. The only evidence that we had was that vibration sense was reduced at the ankles. But in a man of 68 this is not necessarily a sign of anything other than his age. We discharged him in April, oedema-free, and asked him to take chlorthiazide and digitalis. He died at home of heart failure in June.

*Professor Hewer:* Was there any infection anywhere which might account for the amyloid?

*Dr. Harrold:* No sir. His war wounds had developed abscesses but had healed quite quickly.

*Professor Hewer:* Was there ever any bone involvement?

*Dr. Harrold:* No sir, we did not take extensive bone X-rays but there was no clinical evidence of osteomyelitis.

*Dr. Cullen:* Before his first admission he had a change of bowel habit. In view of the subsequent clinical history do you think this could have been due to amyloid?

*Dr. Harrold:* I think it could have been so.

*Professor Hewer:* Did this disturbance of bowel habit continue?

*Dr. Harrold:* No.

*Professor Neale:* Have you any tentative idea of why he went into cardiac failure at this time?

*Dr. Harrold:* I have no idea.

*Professor Neale:* When a person of this age is treated at home for any length of time by chlorthiazide, which we have all agreed has its own potential intrinsic dangers, what precautions do you take to maintain a correct salt balance?

*Dr. Harrold:* When he was in the ward he was on a low salt diet, triamterine, aldactone and chlorthiazide; we checked his serum electrolytes frequently. When he was discharged he was given chlorthiazide alone and allowed salt and given potassium chloride.

*Professor Neale:* I was just wondering what the dangers were, whether there was excessive salt loss or excessive salt retention in a patient with chlorthiazide. I was wondering what the effect of the drug was on the renal tubules.

*Dr. Harrold:* I think the dangers of hyponatraemia are considerable in a person with chronic renal disease, and hepatic disease.

*Dr. Cates:* When we give diuretics to a patient with heart disease we are not treating the heart itself, we are only trying to relieve the symptoms and signs of heart failure. We do not pretend that we are treating his actual heart condition. This man was obviously dying from progressive and untreatable heart disease and I don't think anybody was very surprised when he returned from his home to hospital in gross heart failure despite the fact that had had been taking three powerful drugs at home. The danger of treating oedema due to heart disease too vigorously is not one of producing further oedema but of producing uraemia. Some of us have even been in the embarrassing position of treating a patient with oedema only to find a climbing blood urea and then it has been necessary to treat the patient by re-hydration in order to bring the blood urea down.

*Dr. Partington:* Is there some evidence that this condition may be due to loss of potassium and to alkalosis?

*Dr. Cates:* Yes.

*Dr. Partington:* And there is some evidence, isn't there, that patients on prolonged chlorthiazide may suffer salt loss from the extracellular tissues and the condition becomes irreversible; not responding to administered salt?

*Dr. Cates:* What seems to break down in this event is the cellular mechanism. The body cells lose the ability to push out all the sodium and in the final stages there is an actual influx of sodium into the cells. Obstinate oedema in heart failure is an intriguing clinical problem.

*Dr. Partington:* Do you think that cirrhosis of the liver played any part in this man's oedema? Did you think the finding of this oedema that failed to respond well to the drugs given backs up the evidence that there was liver damage in this case?

*Dr. Cates:* I did not think about it.

*Dr. MacInnes:* In connection with anti-aldosterone compounds and oedema we usually find that they are of little benefit in patients with congestive heart failure. However, in the case of obvious heart failure, say from mitral stenosis with accompanying cardiac cirrhosis and hypoalbuminaemia, we have often seen considerable benefit from the addition of aldactone to the usual diuretic regime.

*Student:* Do you think this was primary or secondary amyloid? I know it is very difficult to tell in these cases.

*Dr. Cates:* We felt it was primary because we had not found any of the causes of secondary amyloidosis.

*Professor Hewer:* I do not see how you could tell in any other way except by finding the cause.

*Dr. Cates:* There are some causes like myelomatosis, that are not infective.

*Professor Neale:* There was cardiomegaly in this case. Were the heart sounds altered very much?

*Dr. Harrold:* There were no murmurs but there was a third sound.

*Professor Neale:* Was the pulse pressure normal?

*Dr. Harrold:* Yes.

*Professor Hewer:* I think we had better have the post mortem now.

*Dr. Cornes:* There is a great deal one might say about this post mortem but I will confine myself to what seemed to be the principal findings. The cause of death was congestive heart failure. The neck veins were distended, there were pleural and peritoneal effusions amounting to some 5 litres altogether. There was pulmonary oedema and congestion of the lungs, liver, kidneys, stomach and intestines. The patient was also jaundiced. The conjunctivae were yellow, and the pleural and peritoneal effusions were deep yellow in colour. The cause of both the jaundice and the heart failure was a diffuse systemic amyloidosis. As I could find no obvious cause for this I presume that this is one of the rare cases of primary amyloidosis.

At post mortem the heart was greatly enlarged and weighed 560 g which is in the usual range of heart weights in patients with amyloid. Not only was the heart enlarged but the right ventricle was dilated and the tricuspid valve measured 16.9 cm in circumference. When the heart was cut open, instead of the walls collapsing in, as they do at post mortem, they remained firm and rigid as though the heart had already been fixed with formalin, and the cut surface of the heart was firm, waxy and had a matt surface. The right ventricle was greatly thickened, to about three times normal thickness, and the left ventricle was also slightly thickened. This thickening was due to amyloid.

In a section of the heart stained with H & E the darker areas represent surviving muscle cells. The amyloid substance, light pink in colour, is all round the cells, in the myocardial fibres and in the wall of the coronary arteries. Another section shows a great mass of amyloid tissue within the myocardium.

In this particular case the amyloid material stained metachromatically with methyl violet. Quite a considerable number of cases of primary amyloidosis fail to show this reaction but I was able to find it in most of the tissues. On the surface of the heart are amyloid deposits around the fat, giving amyloid rings, and the amyloid deposits around the muscle fibres.

A photograph of the subendocardial tissues and myocardium shows colossal deposits of amyloid in the subendocardial tissues (Plate XII) a finding which you get particularly in primary amyloidosis. Sometimes these nodules calcify.

The liver was also very greatly enlarged, weighed 2090 g, and the surface was again waxy to the touch and very firm. The cut surface had an almost nutmeg appearance, presumably due to congestive heart failure. The paler staining areas are largely amyloid substance and they stained positively in the post mortem room with dilute Lugol's iodine. A section from the liver stained with methyl violet shows it to be almost entirely amyloid, and very few surviving liver cells can be seen (Plate XIII). It is not surprising that he was jaundiced.

The spleen was of normal size, and weighed 220 g. The typical picture of the spleen in secondary amyloid is what is known as a sago-spleen, because the amyloid deposits

PLATE XII

*Amyloid deposits in the subendocardial tissues.*
(*H. & E.* × 150.)

PLATE XIII

*Liver.* (*Methyl violet* × 150.)

PLATE XIV

*Tongue. The dark areas represent surviving muscle fibres and the paler areas amyloid. (Methyl violet × 80.)*

PLATE XV

*Intestine showing severe involvement of the lamina propria, muscularis mucosae and sub-mucous blood vessels. (H. & E. × 80.)*

are around the Malpighian bodies giving translucent greyish nodules throughout the spleen. In primary amyloidosis the spleen is either unaffected or diffusely involved, which is what happened in this case. The lymphoid follicles have gone, there is congestion of the sinusoids, and there is a mass of amyloid tissue in between.

Both adrenals were also considerably involved, although macroscopically I had not noticed any abnormality. The adrenal cortex of both glands was almost completely replaced by amyloid substance.

The last slides are just to draw your attention to some of the other features of amyloid. Sometimes, particularly in primary amyloid, you can get a big ulcerated tongue with nodules on it, the so-called scrotal tongue. If you take a biopsy from the tongue you will find amyloid deposits in the muscle (Plate XIV). The trouble with most of the tongue biopsies which I have had in practice has been that they have been very superficial and have missed the muscles so that one has been able to give only an inconclusive answer. If you want to get a good result you have to take a deep biopsy, and this is a painful process and difficult to heal. So a tongue biopsy is helpful, but it can inconvenience the patient!

Amyloid particularly affects small arteries and you get a hyaline appearance of the walls of the smaller vessels. You can make use of this occurrence in biopsy diagnosis when it affects the gastro-intestinal tract (Plate XV). In this particular case the blood vessels of the gastro-intestinal tract were diffusely involved. A section of the intestine shows blood vessels in the submucosa whose walls are a mass of amyloid substance so that a rectal biopsy in this case would have given a positive diagnosis.

*Question:* Did you look at the bone marrow to see if there was any evidence of myeloma?

*Dr. Cornes:* Yes. It was quite normal.

*Dr. Cates:* We must remember that this is a rare disorder; I have only seen three cases of primary amyloid before. Secondary amyloid is associated with myeloma, with infections, with ulcerative colitis, with rheumatoid arthritis and so on, and the amyloid rarely gives rise to a problem in diagnosis. You do not have to think about a differential diagnosis as the main disease steals the picture.

This condition of primary amyloidosis is quite different in several ways. It now seems that quite a number of cases are familial and you can, as with other familial conditions, spot the members of a family who are due to get it in years to come by looking at their plasma proteins and finding an atypical protein band. We did not find any in this man, perhaps because this man was not as far as we know in any of the familial groups of amyloidosis.

Our problem, clinically, is not to wait to diagnose somebody when he has come to the stage of being ready for the pathologist, but to try and spot it much earlier. Perhaps we might have avoided the major operation if we had thought of this diagnosis. Until recently one of the methods of choice was biopsy of the gum but the Israel school, who have collected over 200 cases, have stressed what Dr. Cornes has stressed, that the biopsy site which gives the best results is the rectal mucosa. They found that this was positive in 80 per cent of patients with amyloid disease, compared with the mouth biopsy, in which fewer than 20 per cent were positive.

Clinically the disease can be just about as difficult to diagnose as any of the other diffuse systemic diseases. Puzzling clinical features may be caused by a solitary lump, and blunders are then likely to be made. Thus an amyloid disease in the bronchus may be thought to be a carcinoma, and the patient may be given deep X-rays or amyloid tissue in the lower end of the oesophagus may cause X-ray appearances resembling a carcinoma. The features which are supposed to raise our suspicions of amyloidosis are the association of a large liver and spleen, gastro-intestinal symptoms, heart failure, renal damage and peripheral neuritis.

Better histological techniques of biopsy specimens are said to be responsible for an increase in diagnosis. I wonder whether Dr. Cornes can tell us about the newer ways of staining biopsy material of the rectum which can produce positive findings, for example by using a process involving polarized light.

*Dr. Cornes:* Yes. The latest method introduced by Canadian workers employs thioflavine T (Vasser and Culling, 1959) and you look at the sections with ultra-violet light; the amyloid tissue fluoresces a golden colour. The only other tissue that does this is mucin in mucophages according to the nature of the mounting medium used. I have never used it myself but I am told by people who have used it that sometimes when the congo red and methyl violet tests are doubtful this test has been positive (Hobbs and Morgan, 1963). This stain can also give a negative result when congo red stains are positive.

*Dr. Norman:* In Alzheimer's disease it has been shown that the senile plaques and neutrophils contain an amyloid component which is birefringent after staining with congo red. Is this birefringence a valuable sign in other forms of amyloidosis?

*Dr. Cornes:* I don't think anyone knows the answers to those questions. Birefringence is certainly a variable feature.

*Dr. McInnes:* Have steroids any effect on the course of this disease?

*Dr. Harrold:* In animals amyloid disease can be produced by steroids and a high protein diet.

*Question:* I wonder if the primary and secondary amyloid are the same substance?

*Professor Hewer:* The trouble is one does not really know exactly what the substance is in either case—it is complex and variable.

*Dr. Cates:* There are some recent fascinating studies from Israel (Sohar *et al*, 1963). Amongst the Ashkenazy Jews, Jews who live around and about the Mediterranean, there is a familial disease of long continued fever of unknown origin called Mediterranean fever. It may eventually be complicated by the presence of amyloidosis. The puzzle is that the amyloid which complicates this familial fever, genetic in origin, has a distribution and other characterisitics you see in secondary amyloid. The distribution of amyloid is divided into two groups: one is usually classified as peri-reticular and the other one peri-collagen. The puzzle is why this familial primary amyloid should have a secondary type of distribution.

*Post-Script*

In the post mortem records of the Department of Pathology of the University of Bristol covering some 8,500 post mortems there are 49 cases of amyloidosis, of which 10 are primary cases.

REFERENCES

Hobbs, J. R. and Morgan, A. D. (1963) *J. Path. Bact.*, **86**, 437.
Sohar, E., Gafni, J., Blum, A., Pras, M. and Heller, H. (1963). *Quart. J. Med., N.S.* **32**, 211.
Vassar, P. S. and Culling, C. F. A. (1959). *Arch. Path.*, **68**, 487.

## Figures and Tables

**PLATE XII f1:**
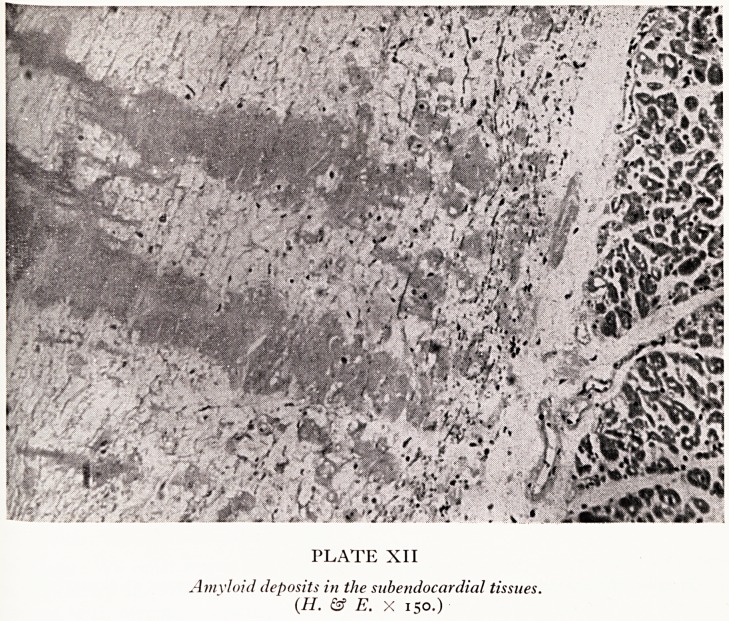


**PLATE XIII f2:**
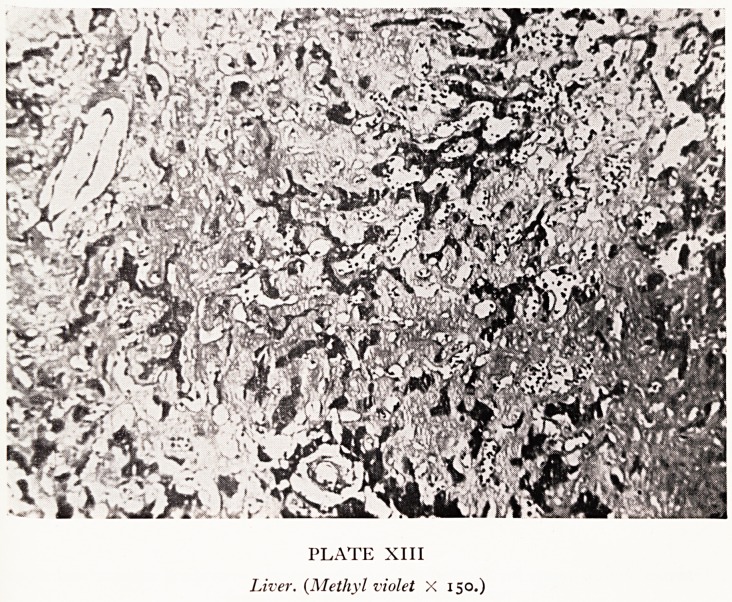


**PLATE XIV f3:**
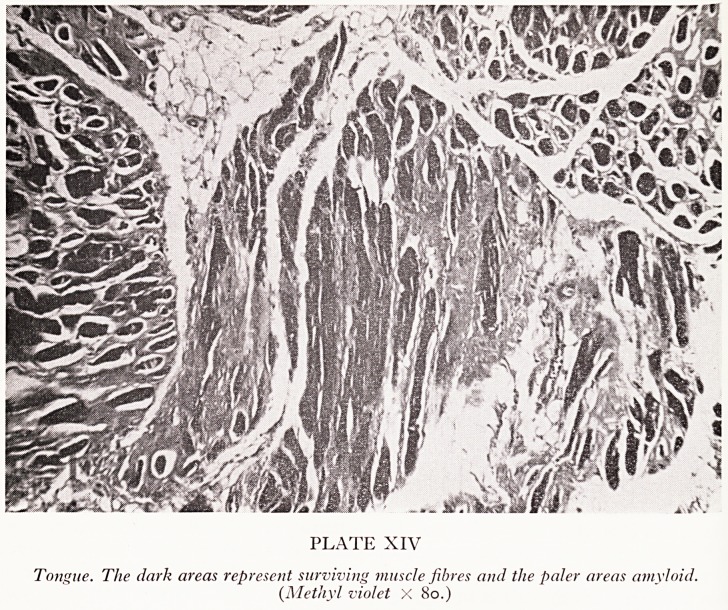


**PLATE XV f4:**